# Production of bioactive chicken (*Gallus gallus*) follistatin-type proteins in *E. coli*

**DOI:** 10.1186/s13568-015-0142-3

**Published:** 2015-08-25

**Authors:** Sang Beum Lee, Sung Kwon Park, Yong Soo Kim

**Affiliations:** Department of Human Nutrition, Food and Animal Sciences, University of Hawaii, 1955 East-West Rd., Honolulu, HI 96822 USA; Department of Food Science and Technology, Sejong University, Seoul, 143-747 Korea

**Keywords:** Follistatin, Follistatin-type protein, *E. coli* production, Recombinant protein, Ligand selectivity

## Abstract

Follistatin (FST) is a cysteine-rich autocrine glycoprotein and plays an important role in mammalian prenatal and postnatal development. FST binds to and inhibit myostatin (MSTN), a potent negative regulator of skeletal muscle growth, and FST abundance enhances muscle growth in animals via inhibition of MSTN activity. The objective of this study was to produce biologically active, four chicken FST-type proteins in an *Escherichia coli* expression system. Gibson assembly cloning method was used to insert the DNA fragments of four FST-type proteins, designated as FST288, NDFSD1/2, NDFSD1, and NDFSD1/1, into pMALc5x vector downstream of the maltose-binding protein (MBP) gene, and the plasmids containing the inserts were eventually transformed into *Shuffle E. coli* strain for protein expression. We observed a soluble expression of the four MBP-fused FST-type proteins, and the proteins could be easily purified by the combination of amylose and heparin resin affinity chromatography. MBP-fused FST-type proteins demonstrated their affinity to anti-FST antibody. In an in vitro reporter gene assay to examine their potencies and selectivities to different ligands (MSTN, GDF11, and activin A), the four FST-type proteins (MBP-FST288, MBP-NDFSD1/2, MBP-NDFSD1, and MBP-NDFSD1/1) showed different potency and selectivity against the three ligands from each other. Ligand selectivity of each FST-type proteins was similar to its counterpart FST-type protein of eukaryotic origin. In conclusion, we could produce four FST-type proteins having different ligand selectivity in *E. coli*, and the results imply that economic production of a large amount of FST-type proteins with different ligand selectivity is possible to examine their potential use in meat-producing animals.

## Introduction

Improving the efficiency of meat-animal production is crucial to a sustainable supply of quality proteins to humans with minimal environmental footprints. Muscle growth efficiency is probably one of the main determinants of meat production efficiency, and some studies indicate that enhancing skeletal muscle growth improves the efficiency of feed utilization (Bailey et al. 1966; Webster 1977), resulting in improved efficiency of meat-animal production. Technologies positively modulating muscle growth process would contribute to enhancing meat-production efficiency. Recent studies have shown that myostatin (MSTN), a member of the transforming growth factor-β superfamily (TGF-β), is the most potent negative regulator of skeletal muscle growth (Lee 2004), implying that suppression of MSTN activity would be a strategy to improve skeletal muscle growth. In lab animals, many studies have indeed demonstrated that skeletal muscle growth can be enhanced by suppressing MSTN activity (Joulia-Ekaza and Cabello 2007; Lee 2004; Rodgers and Garikipati 2008). It has also been shown that *in*-*ovo* suppression of MSTN activity via anti-MSTN antibodies significantly improves post-hatch skeletal muscle growth of broilers (Kim et al. 2006), demonstrating that inhibition of MSTN activity is a viable strategy to enhance muscle growth in meat-producing animals.

One of such molecules suppressing MSTN activity is follistatin (FST), a cysteine-rich autocrine glycoprotein that plays an important role in mammalian prenatal and postnatal development. FST was initially identified as an inhibitor of follicular stimulating hormone via binding to activin, thus thought that the biological activity of FST was restricted to the reproductive system (Robertson et al. 1987; Ueno et al. 1987). Further investigations, however, revealed that FST binds to multiple members of the TGF-β superfamily, and that biological activities of this protein encompass multiple organ systems, including bone, skeletal muscle, and liver (DePaolo et al. 1991; Phillips and de Kretser 1998). In chicken pectoral muscle cell cultures, FST enhanced muscle cell development (Link and Nishi 1997). FST’s inhibition of MSTN binding to its receptors has been demonstrated in vitro (Lee and McPherron 2001), supporting that the enhancement of muscle cell development by FST was probably due to FST’s suppression of MSTN. Subsequently, various studies have shown that the abundance of FST or FST fragment in muscle via transgenesis, injection of expression plasmid, or single administration of FST gene via adeno-associated virus delivery system significantly increased skeletal muscle mass/strength (Gilson et al. 2009; Haidet et al. 2008; Kota et al. 2009; Lee and McPherron 2001; Nakatani et al. 2008). Interestingly, the muscle mass increase in transgenic mice overexpressing FST was significantly greater than that in MSTN null mice (Lee and McPherron 2001), and recent results suggest that enhancement of muscle growth by FST is not only via MSTN suppression but also involves activin-dependent mechanisms (Gilson et al. 2009; Lee 2007; Lee et al. 2010). Transgenic rainbow trout overexpressing FST exhibited dramatic muscularity (Medeiros et al. 2009). These results together indicate that FST would be a potential agent to improve skeletal muscle growth in agricultural animals, as well as, to treat skeletal muscle atrophic disorders in humans.

**Fig. 1:**
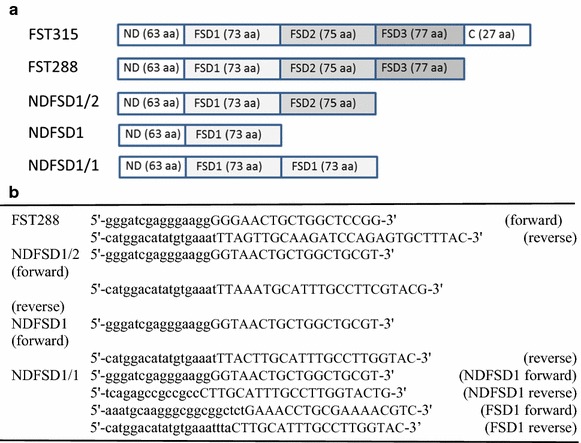
Different FST-type proteins (**a**) and Gibson assembly primer sets for four different FST-types (**b**). The sequences in *small capital* indicate overlap sequence part and the sequences in all capital indicate primer sequence part used for Gibson assembly cloning

In a previous study, we were successful in producing bioactive full sequence of chicken FST (FST315) in an *E. coli* system using maltose binding protein (MBP) as a fusion partner (Lee et al. 2014), illustrating the potential of economic production of FST for application in meat-producing animals. FST is a multi-domain protein consisting of 5 domains (Fig. 1a), and FST-type proteins containing different FST domains have differential ligand suppressing activities (Cash et al. 2012; Nakatani et al. 2011; Schneyer et al. 2008; Sidis et al. 2006). The ligand selectivity of FST-type proteins is an important property for FST-type proteins to be used in animals for improving skeletal muscle growth with minimal undesirable side effects that may arise from FST’s interaction with other ligands other than MSTN. It was, thus, contended that producing different FST-type proteins in an *E. coli* system and determining their bioactivities and ligand selectivity would contribute to examining their potentials in meat animal production. Therefore, the objective of this study was to produce biologically active, four different chicken FST-type proteins in an *E. coli* expression system. Results of this study show that bioactive, four FST-type proteins can be produced in an *E. coli* system, and their ligand selectivity is different from each other, but the ligand selectivity of each FST-type protein is similar to its counterpart FST-type protein of eukaryotic cell origin.

## Materials and methods

### Cloning of four FST-type proteins into an expression vector and transformation of expression vectors

Since we previously used pMALc5x vector (New England Biolabs, MA, USA) in cloning for expression of bioactive chicken FST315 in an *E. coli* system (Lee et al. 2014), the same system was used in cloning four different FST-type proteins. The four FST-type proteins included FST288, NDFSD1/2, NDFSD1/1, and NDFSD1 (Fig. 1a). Gibson assembly cloning method (Gibson et al. 2009) was used to insert the DNA fragments of four FST-type proteins into pMALc5x vector separately. Gibson assembly primers for FST288 (GenBank Accession No. KT336491), NDFSD1/2 (GenBank Accession No. 336492), NDFSD1/1 (GenBank Accession No. 336493), and NDFSD1 (GenBank Accession No. 336494) fragments were synthesized (Fig. 1b), and inserts were prepared by PCR amplification using the Q5 High-Fidelity PCR kit (New England Biolabs) and chicken FST315 cDNA (Lee et al. 2014) as a template. PCR products were separated by agarose gel electrophoresis, and fragments were excised and purified before use in DNA assembly reaction with XmnI-linearized pMALc5x vector using Gibson Assembly^®^ Cloning kit (New England Biolabs). Each assembly reaction contained approximately 100 ng of insert and 50 ng of the expression vector and incubated at 50 °C for 30 min following the manufacturer’s protocol. After the assembly reaction, the reaction mix was transformed into NEB 5-alpha competent *E. coli* strain (New England Biolabs). After an overnight growth at 37 °C, the pMAL-c5x plasmids containing respective inserts were extracted using a plasmid extraction miniprep kit (Promega) to confirm correct insertion by colony PCR.

### Cytoplasmic expression of four FST-type proteins

*Shuffle E. coli* (New England Biolabs) were transformed with pMALc5x-FST288, pMALc5x-NDFSD1/2, pMALc5x-NDFSD1, and pMALc5x-NDFSD1/1 plasmids. After confirmation of correct insertion by colony PCR, 5 mL Luria–Bertani (LB) (1.2 % tryptone, 0.6 % yeast extract and 0.8 % NaCl) medium containing 100 μg/mL ampicillin and 0.2 % glucose were inoculated individually with the *Shuffle E. coli* harboring the inserts, and grown overnight at 30 °C with vigorous shaking. Then, 5 mL of the overnight cultures were transferred into 1 L fresh LB medium containing ampicillin in 2 L flask. When the culture reached to an optical density of 0.3–0.4 Å (600 nm) at 30 °C, the cultures were transferred to 4 °C for protein expression induced by adding Isopropyl β-d-1-thiogalactopyranoside (IPTG) to a final concentration of 0.4 mM under vigorous shaking. After induction for 8 days, *E*.*coli* pellet was harvested by centrifugation at 4000*g* for 10 min at 4 °C. Each gram (wet weight) of the cell pellet was resuspended in 5 mL of affinity column buffer (20 mM Tris–Hcl, 200 mM NaCl, 1 mM EDTA, pH 7.4) containing the Complete Mini Protease Inhibitor cocktail tablet (Roche, Mannheim, Germany). Two microliter of lysozyme (50 μg/mL) and 2 μL of DNase I (2500 units/ml) were added per 1 mL column buffer. The resuspended cell solution was lysed by sonication in short pulses of 15 s for 10 min in ice water bath. The soluble and insoluble fractions were prepared by centrifugation at 10,000*g* for 20 min at 4 °C. For each sample, the supernatants (soluble fraction) were collected, and the same volume of column buffer was used to resuspend the pellets (insoluble fraction). Total, soluble and pellet fractions were analyzed by SDS-PAGE to examine the presence of MBP-fused recombinant proteins (MBP-FST288, MBP-NDFSD1/2, MBP-NDFSD1, and MBP-NDFSD1/1).

### Sodium dodecyl sulfate polyacrylamide gel electrophoresis (SDS-PAGE)

SDS-PAGE was performed with gels containing l2.5 % polyacrylamide and 0.1 % SDS following the procedure of Laemmli (1970). Samples were mixed with 3X loading buffer which were under reducing conditions. Before loading the sample onto the SDS-PAGE gel, samples were boiled at 100 °C for 5 min.

### Amylase affinity purification of MBP-fused FST-type proteins

Supernatant cell extracts were diluted with affinity column buffer in a 1:5 ratio, and filtered through 0.45 μm filter, then was loaded into an amylose resin column equilibrated with 100 mL of column buffer. After loading, the pass-through was collected at a rate of 0.5 ml/min and washed with 100 mL of column buffer. Proteins bound to the column were then eluted with elution buffer (column buffer with 10 mM maltose) at a flow rate of 0.5 ml/min. 5 mL fractions were collected during elution, and the absorbance was monitored at 280 nm. After SDS-PAGE analysis of the presence of recombinant protein in fractions, fractions containing recombinant proteins were pooled.

### Heparin affinity purification of MBP-fused FST-type proteins

For further purification of amylose affinity-purified MBP-fused proteins, the pooled elutions were subjected to heparin affinity column (Bio-Rad, CA, USA) previously equilibrated with column buffer. The pass-through was collected at a rate of 1 ml/min. The column was then washed with 100 mL of column buffer. Proteins bound to the column were then eluted with elution buffer (column buffer with 1 M NaCl). After SDS-PAGE analysis of the presence of recombinant protein in fractions, fractions containing recombinant proteins were pooled, followed by dialysis in phosphate buffered saline solution.

### Western-blot analysis

Affinity-purified MBP-fused proteins were subjected to a 12.5 % SDS-PAGE, followed by a transfer onto a polyvinylidene fluoride (PVDF) membrane. The membrane was blocked for 1 h at room temperature with 5 % non-fat dry milk in Tris-buffered saline with 0.1 % Tween-20 (TTBS). The membranes was incubated with the goat anti-FST (1:5,000 in TTBS, R&D Systems, MN, USA) overnight at 4 °C. The membrane was then washed three times (10 min for each wash) with TTBS, followed by incubation with 1:10,000 alkaline phosphatase-conjugated anti-goat IgG (Sigma, MO, USA) in TTBS for 1 h. After washing, the membrane was developed using BCIP/NBT substrate (nitrobluetetrazolium and bromo-chloro-indolyl phosphate from Sigma).

### Bioactivity test of MBP-fused FST-type proteins by pGL3-(CAGA)_12_ Luc-luciferase reporter system

The capacities of MBP-FST288, MBP-NDFSD1/2, MBP-NDFSD1, and MBP-NDFSD1/1 to suppress the bioactivity of three FST-binding proteins (MSTN, GDF11, and activin A, all from R&D Systems) were examined by a procedure that was used in examining the bioactivity of MBP-fused chicken FST315 (Lee et al. 2014). Briefly, cells stably transformed with pGL3-(CAGA)_12_ luciferase reporter construct (Cash et al. 2012) were seeded in a 96 well plate for 24 h at 37 °C with 5 % CO_2_. The medium was replaced with 100 μL serum-free DMEM containing 1 nM MSTN, GDF11 or activin A plus various concentrations of FST-type proteins, then incubated for 24 h. After removing the medium, Bright-Glo luminescence substrate (Promega) was added, and luminescence was measured. The  % inhibition of MSTN, GDF11 or activin A activity was calculated by the following formula: % inhibition = (luminescence at 1 nM MSTN, GDF11 or activin A-luminescence at each ligand concentration) × 100/(luminescence at 1 nM MSTN, GDF11 or activin A-luminescence at 0 nM MSTN, GDF11 or activin A). The MSTN-, GDF11- or activin A-inhibitory activity was analyzed by regression analysis using Prism 5 program (Graphpad, CA, USA). To examine the differences in MSTN-, GDF11- or activin A-inhibitory capacity of these proteins, IC_50_ (ligand concentration inhibiting 50 % of MSTN, GDF11 or activin A activity) values were estimated using a non-linear regression model defining dose response curve. The equation for the model was as follows: Y: Bottom + (Top − Bottom)/[1 + 10^(X − LoglC_50_)], where Y is % inhibition, Bottom is the lowest value of % inhibition, Top is the highest value of % inhibition, and X is Log ligand concentration IC_50_ values were analyzed by ANOVA (Analysis of Variance) using the same program.

## Results

### Cytoplasmic expression of MBP-fused FST-type proteins in *Shuffle E. coli* system

**Fig. 2:**
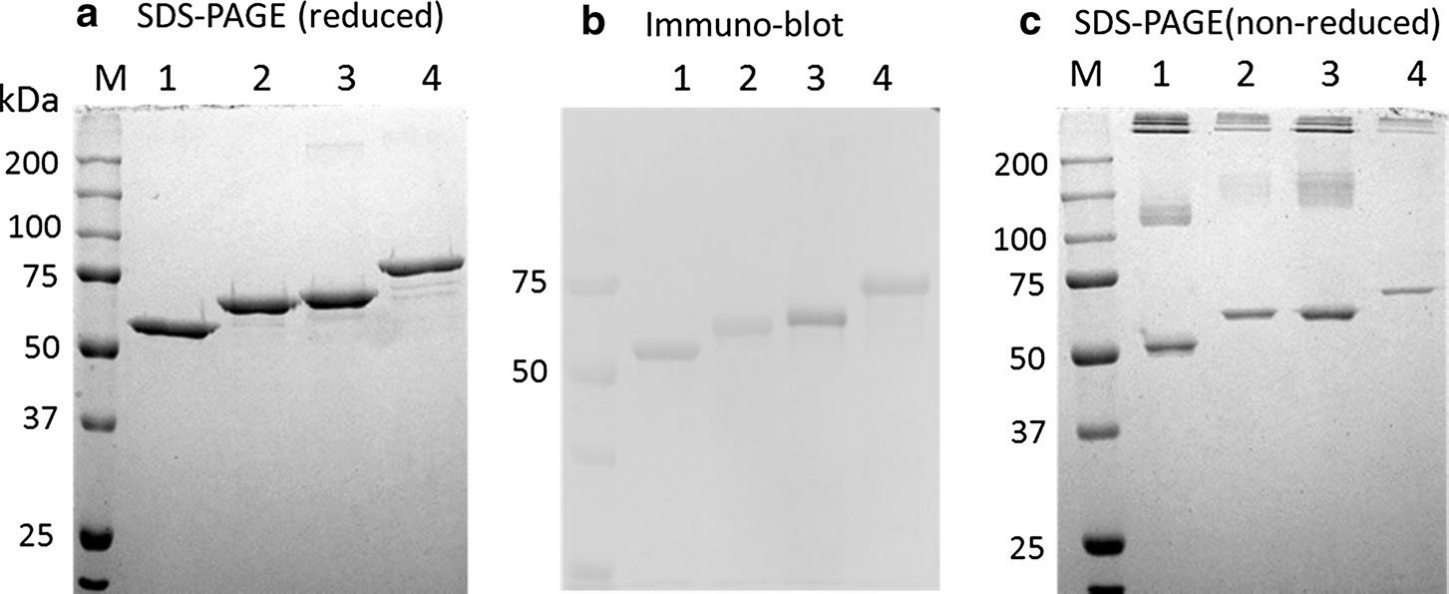
SDS-PAGE and Western blot analysis of four FST-type proteins containing MBP as a fusion partner. Purified protein samples were subjected to 12.5 % SDS-PAGE either in reduced (**a**) or non-reduced (**c**) conditions and stained with Coomassie blue. Proteins fractionated by SDS-PAGE were transferred onto polyvinylidene fluoride (PVDF) membrane electrophoretically, followed by immunoblotting against polyclonal anti-human FST (1:200) antibodies (**b**). Lanes 1, 2, 3 and 4 indicate MBP-NDFSD1, MBP-NDFSD1/2, MBP-NDFSD1/1, and MBP-FST288, respectively

In our previous study (Lee et al. 2014), we observed that induction at 4 °C for 8 days resulted in a higher yield of affinity-purified, MBP-fused chicken FST315 than 8 h induction at 25 °C even though the yield of soluble forms of the recombinant protein was much higher at 25 °C induction, probably due to an improvement in proper folding at lower temperature. Thus, we expressed four FST-type proteins (MBP-FST288, MBP-NDFSD1/2, MBP-NDFSD1, and MBP-NDFSD1/1) at 4 °C for 8 days, and purified the proteins. Like the MBP-fused chicken FST315, all four FST-type proteins were expressed in a soluble form (Data not shown). When soluble fractions of these proteins were purified using a combination of amylose and heparin affinity chromatography, the purification appeared to be more than 90 %, and the locations of each form of FST in reduced SDS-PAGE gel were at the expected size (Fig. 2a). Western blot analysis showed that the four FST-type proteins had affinity to anti-FST antibody (Fig. 2b), confirming the identity of each proteins. When the proteins were subjected to SDS-PAGE in non-reduced condition (Fig. 2c), presence of some aggregates was observed in all four FST-type proteins with almost equal proportion at around 50 %.

The yield of amylose/heparin affinity-purified MBP-FST288, MBP-NDFSD1/2, MBP-NDFSD1/1, and MBP-NDFSD1 were 7.5, 15.8, 14.5 and 19.8 mg/L culture, respectively (Table 1). In our previous study, the yield of amylose/heparin affinity purified MBP-FST315 was 5.8 mg/L culture (Lee et al. 2014). It, thus, appears that the yield of MBP-fused FST-type protein is negatively related to the size of molecules.

**Table 1:** Yields of various FST-type proteins after combined affinity purification of amylose and heparin resins

FST-type proteins	mg/L
MBP-FST288	7.5
MBP-NDFSD1/2	15.8
MBP-NDFSD1/1	14.5
MBP-NDFSD1	19.8

Protein concentration was measured by Lowry method using BSA as a standard. The protein recovery was calculated as the mean ± SEM from purifications of a triplicate of 1 L culture of each FST-type proteins

### Bioactivity of MBP-fused FST-type proteins

**Fig. 3:**
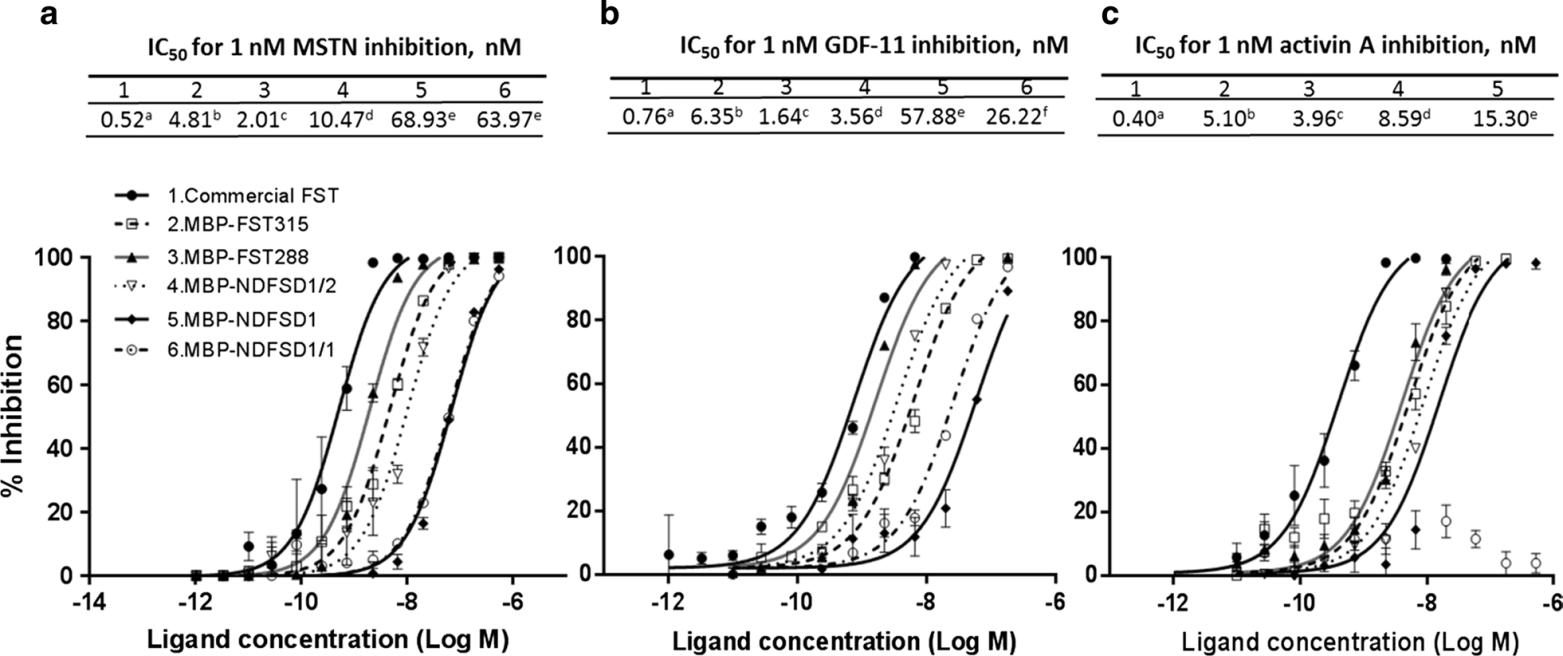
Inhibition of MSTN, GDF11 or activin A activities by various FST-type proteins. HEK293 cells stably expressing (CAGA)_12_-luciferase gene construct were seeded on a 96-well culture, and grown for 24 h in DMEM with 10 % fetal calf serum, antibiotic and antimycotic. Medium was removed, and MSTN (1 nM), GDF11 (1 nM) or activin A (1 nM) plus various concentrations (180–0 nM) of FST-type proteins in DMEM were added to each well, followed by incubation for 24 h. Medium was removed, and luminescence substrate was added, followed by luminescence measurement. The error bars represent ± SEM (n = 6)

The abilities of MBP-FST288, MBP-NDFSD1/2, MBP-NDFSD1, and MBP-NDFSD1/1 to suppress MSTN, GDF11, and activin A were examined using pGL3-(CAGA)_12_ Luc-luciferase reporter assay (Fig. 3), and their potencies were compared each other, as well as to commercial recombinant human FST produced in eukaryotic cells (rhFST315/CHO, R&D Systems) or to MBP-FST315. It has been observed in our previous study that the presence of MBP as a fusion partner had no influence on the MSTN- or activin A-inhibitory capacity of FST315 (Lee et al. 2014). The removal of aggregates appeared in non-reduced condition of SDS-PAGE by gel-filtration did not significantly affect the MSTN- or activin A-inhibitory capacity of FST315 (Lee et al. 2014). Therefore, in measuring the bioactivities of FST-type proteins, we used amylose/heparin affinity-purified proteins without further purification.

Figure 3a shows the inhibition of 1 nM MSTN by different forms of MBP-fused FST. The IC_50_ values of MBP-FST288, MBP-NDFSD1/2, MBP-NDFSD1, and MBP-NDFSD1/1 to suppress MSTN were 2.01, 10.47, 68.93, and 63.97 nM, respectively, and these values were significantly different from each other except the values between MBP-NDFSD1 and MBP-NDFSD1/1. The IC_50_ values of these proteins for suppression of MSTN were significantly higher than that of rhFST315/CHO (0.52 nM). The IC_50_ value of MBP-FST288 (2.01 nM) for suppression of MSTN was significantly lower than that of MBP-FST315 (4.81 nM).

Figure 3b shows the inhibition of 1 nM GDF11 by different FST-type proteins. The IC_50_ values of MBP-FST288, MBP-NDFSD1/2, MBP-NDFSD1, and MBP-NDFSD1/1 to suppress GDF11 were 1.64, 3.56, 57.88, and 26.22 nM, respectively, and these values were significantly different from each other. The IC_50_ values of these proteins for suppression of GDF11 were significantly higher than that of rhFST315/CHO (0.52 nM). MBP-FST288 had lower IC_50_ value than MBP-FST315 (1.64 vs 6.35 nM).

Figure 3c shows the result of the inhibition of 1 nM activin A by different FST-type proteins. The IC_50_ values of MBP-FST288, MBP-NDFSD1/2, and MBP-NDFSD1 to suppress activin A were 3.96, 8.59 and 15.30 nM, respectively. MBP-NDFSD1/1 did not show its capacity to suppress activin A activity. The IC_50_ value of rhFST315/CHO (0.40 nM) was significantly lower than those of MBP-fused FST-type proteins.

## Discussion

FST is a multi-domain glycoprotein consisting of 5 domains, including N-terminal domain followed by three FST domains (FSD1-3) and C-terminal domain (Shimasaki et al. 1988). Three isoforms of FST, including FST315, FST303, and FST288, have been identified in vivo (Schneyer et al. 2004). FST315 encompasses all domains, and FST288 is lacking the C-terminal domain, and FST303 arises from proteolytic cleavage of the FST315 C-terminal tail (Sugino et al. 1993). These isoforms have shown locational compartmentalization and different biological roles in vivo (Kimura et al. 2010; Schneyer et al. 2004). Studies, which analyzed the importance of the different domains and domain arrangement of FST-type proteins on ligand binding, have shown that FST-derived peptides containing different FST domains have differential MSTN, activin and GDF11 suppressing activities (Cash et al. 2012; Nakatani et al. 2011; Schneyer et al. 2008; Sidis et al. 2006), illustrating the potential to obtain selective antagonists against a specific ligand. The different FST-type proteins used in those studies were all eukaryotic origin. As compared to *E. coli* systems, the use of eukaryotic system is in general more costly in producing recombinant proteins, limiting research with the recombinant proteins on meat-producing animals. Recently, we expressed bioactive chicken FST315 in a soluble form in *E. coli* using MBP as a fusion partner (Lee et al. 2014), and in this study, we were also able to produce bioactive four chicken MBP-fused FST-type proteins, including MBP-FST288, MBP-NDFSD1/2, MBP-NDFSD1, and MBP-ND-FSD1/1. Our results also showed that the production yield increases with the decrease in the size of FST-type proteins.

We examined the capacities of these four FST-type proteins to suppress the activities of MSTN, GDF11, and activin A, which share a common core of signaling components, including type I and type II activin receptors and Smad2 and 3 phosphorylation. In our previous study (Lee et al., 2014), we observed that the potency of chicken MBP-FST315 to suppress MSTN or activin was lower than mammalian-derived human FST, and cleavage of MBP from MBP-FST315 did not change the potency, indicating that MBP fusion does not affect MBP-fused FST bioactivity. It is, thus, possible that the lower potency of MBP fused chicken FST315 compared to mammalian-derived human FST was due to a sequence difference between chicken and human FST. Since MBP fusion showed no effect on FST bioactivity, the bioactivities of four FST-type proteins were examined without MBP removal.

The examination of the capacity of these FST-type proteins to suppress three different ligands (MSTN, GDF11, and activin A) revealed that their ligand selectivity is different from each other. The IC_50_ values of MBP-FST288 to suppress MSTN, GDF11, and activin A were significantly lower than those of MBP-FST315, indicating that MBP-FST288 is more potent in suppressing the three ligands than MBP-FST315. This result is in agreement with previous studies, in which FST288 of eukaryotic origin had a higher affinity or inhibitory potency for various ligands compared with FST315 of eukaryotic origin (Hashimoto et al. 2000; Inouye et al. 1991; Sidis et al. 2006; Sugino et al. 1993). It is, thus, indicated that the N-terminal presence of MBP in FST288 did not have much influence on the interaction of this molecule with the three ligands.

The potencies of MBP-NDFSD1/2 to suppress MSTN, GDF11 and activin A were two-five fold lower compared with MBP-FST288, indicating that the FSD3 of FST contribute to the binding of FST to its ligands. In support of the result, Cash et al. (2012) recently reported that FSD3 provides affinity to ligand and stability on perturbations in the ND and FSD2 probably through the interaction of FSD3 of one FST molecule with the ND of the other FST molecule. It is generally regarded that a continuous sequence comprising ND followed by FSD1 and 2 is essential for activin binding and bioactivity (Keutmann et al. 2004). The crystal structure of FST288:activin complex has shown that the type II receptor binding site is blocked by both FSD1 and FSD2 (Thompson et al. 2005). Results by Schneyer et al. (2008) showed that activin binding and neutralization are mediated primarily by FDS2, whereas MSTN binding is more dependent on FSD1, such that deletion of FSD2 or adding an extra FSD1 in place of FSD2 create MSTN antagonists with vastly reduced activin antagonism. In support of the crucial role of FSD2 for activin inhibition, Keutmann et al. (2004) reported that eukaryotic cell-originated FST comprising only ND plus FSD1 had no biological activity at a dose 1000 fold higher than that eliciting a significant activity from FST288. In the current study, the potency of MBP-NDFSD1 to suppress the above three ligands significantly lower than MBP-FST315, MBP-FST288, or MBP-NDFSD1/2, supporting the role of FSD2 in ligand binding. However, in contrast to the result of Keutmann et al. (2004) and Schneyer et al. (2008), our result shows that the presence of FSD2 is more crucial for inhibition of MSTN or GDF11 than the inhibition of activin A because the potency decreases of MSTN and GDF11 from MBP-NDFSD1/2 to MBP-NDFSD1 were about 6 fold and 15 fold, respectively, while the decrease was only 2-fold for activin A. Given that the FST-type proteins used in this study contained MBP as a fusion partner with no glycosylation due to its *E. coli* origin, it is speculated that either the presence of MBP and/or lack of glycosylation potentially affected the interaction between FSD1 and activin A, MSTN or GDF11.

It has been recently reported that FST-NDFSD1/1 had an affinity for MSTN, but not for activin A (Cash et al. 2012; Nakatani et al. 2008; Schneyer et al. 2008), indicating that NDFST1/1 have a potential as an agent to improve muscle growth via specific MSTN inhibition with minimal effect on activin activity. Like the NDFSD1/1 of eukaryotic origin, the MBP-NDFSD1/1 showed that it did not inhibit activin A activity, but inhibited MSTN activity with similar potency to MBP-NDFSD1. MBP-NDFSD1/1 also suppressed the GDF11 activity. In agreement with this result, the NDFSD1/1 of eukaryotic origin has also shown to inhibit GDF11 (Schneyer et al. 2008). Interestingly, the potency of MBP-NDFSD1/1 to suppress GDF11 was almost 2.5 fold higher compared with MBP-NDFSD1. The results together indicate that MBP-NDFSD1/1 can suppress MSTN activity without much effect on activin, but it can suppress GDF11 activity, thus potential side effects arising from GDF11 inhibition by NDFSD1/1 need to be considered in using NDFSD1/1 as an agent to improve skeletal muscle growth via MSTN inhibition. Furthermore, FST315 and FST288 have been shown to bind to other TGF-beta superfamily member proteins, such as BMP-6 and MBP-7 (Sidis et al. 2006), and current study did not examine the binding of FST-type proteins to other TGB-beta superfamily member proteins. Thus, the binding of FST type proteins to other TGF-beta superfamily member proteins also needs to be examined before consideration of these proteins for an agent to improve skeletal muscle growth or for other therapeutic potentials. In summary, the current study demonstrated that bioactive chicken FST-type proteins can be produced in an *E. coli* system. Current results also show that the ligand selectivity of four different FST-type proteins is different from each other, but the ligand selectivity of each FST-type protein is similar to its counterpart FST-type protein of eukaryotic origin.
